# Postoperative Hemoglobin Drop and the Associated Factors among Elective Orthopedic Surgeries in Northern Tanzania

**DOI:** 10.1155/2024/4145592

**Published:** 2024-05-24

**Authors:** Abdel Ashir, Elifuraha G. Maya, Shahnoor Ruhulamin Saiyed, Taher Muslim Alimohamed, Mubashir Alavi Jusabani, Kulthum A. Abdel, Abid M. Sadiq, Ali Mohamed Ali, Faiton Ndesanjo Mandari

**Affiliations:** ^1^Faculty of Medicine, Kilimanjaro Christian Medical University College, Moshi, Tanzania; ^2^Department of Orthopedics and Trauma, Kilimanjaro Christian Medical Centre, Moshi, Tanzania; ^3^Faculty of Medicine, Catholic University of Health and Allied Sciences, Mwanza, Tanzania

## Abstract

**Background:**

Having an estimated level of Hb drop in different orthopedic surgeries would help plan for surgery from pre- to postoperative periods. The aim of this study was to assess the Hb drop and the associated factors during the intraoperative period among elective orthopedic surgeries.

**Methods:**

This was an analytic cross-sectional study conducted between October 2022 and March 2023, which included all patients admitted for elective orthopedic surgery who met the inclusion criteria. Data were collected before and after the patient was operated on. Information was analyzed using *t*-tests and ANOVA to establish the statistical significance of the Hb drop.

**Results:**

A total of 195 participants were enrolled. The majority of the participants were male (62.1%), with the main etiology of symptoms being motor traffic accidents (31.8%). The most affected site was the femur (36.4%), followed by the spine (23.6%). The highest mean Hb drop was in total hip replacement surgeries (4.19 g/dL), with the overall mean Hb drop being 2.75 g/dL. A statistically significant difference was identified in diathermy use, duration of surgery, and patients with chronic illnesses.

**Conclusion:**

With a mean Hb drop of 2.75 g/dL, the application of diathermy and surgeries with shorter durations resulted in a reduced Hb drop. These factors should be incorporated to minimize the drop in Hb in orthopedic surgeries. Accounting for differences in surgeries, there should not be delays in patients who have a preoperative Hb level that can sustain the mean Hb drop recorded in the study.

## 1. Introduction

Postoperative hemoglobin (Hb) levels are most often considered to be reflective of perioperative blood loss [1]. Preoperative requests for blood for elective surgery are common due to worse-case scenarios and overestimating anticipated blood loss, which is hardly required. This may cause a strain on valuable resources both in technician effort and biochemical reagents, especially in resource-limited settings [2]. In most orthopedic surgeries, blood loss is characterized by slow, continuous hemorrhage characteristic of muscle stripping, subperiosteal dissection, and osteotomy [3].

The preoperative assessment is the best moment to decide the most appropriate strategy to minimize the risk of blood transfusion based on the type of surgery, the patient's health status, the estimated time to surgery, and available blood-saving techniques. Possible risk factors for receiving perioperative blood transfusions include the surgeon's skill, type of surgery, female gender, low body mass, low preoperative Hb level, bleeding disorders, drugs that alter hemostasis, and comorbidities, especially cardiovascular or respiratory disease [4].

Variations in transfusion rates may be due to multiple factors, including differences in surgical and anesthetic techniques, differing opinions on the minimal threshold level of Hb, preoperative anemia, and the lack of availability of transfusion protocols. This may reflect doubt about the relative risks and benefits of transfusion and the different perceptions of the value of minimizing blood loss and subsequent transfusion [2]. The most common orthopedic procedures done in northern Tanzania are femur fixations, followed by tibia and/or fibula fixations, ankle reconstructions, and lastly, spine and upper limb surgeries [5].

Perioperative blood management relies on minimizing blood loss through hypotensive anesthesia measures, mechanical hemostasis devices, hemodilution, reinfusion devices, and pharmacologic antifibrinolytic agents [6]. The use of diathermy is a practical way of minimizing blood loss during surgery. Hemodilution has also been shown to be a cost-effective way of decreasing blood loss. Good anesthetic technique reduces episodes of hypertension and tachycardia due to sympathetic overactivity by ensuring adequate anesthesia and analgesia [3].

The surgical approach poses significant considerations that must be accounted for in the anesthetic plan. Both general and regional anesthesia can be used to care for patients undergoing elective orthopedic surgery. When compared to general anesthesia (GA), patients undergoing major orthopedic surgeries with regional anesthesia experience reduced blood loss, the need for transfusions, and postoperative pain [7]. Regional anesthesia is also associated with a decreased risk of stroke, embolic, and cardiac risk [8].

Quite a number of surgeries are postponed every day due to the lack of blood in the hospital. This tends to extend hospital stays for patients who could be operated on and discharged as early as possible. Extended stays expose the patient to a number of hospital factors, for example, hospital-acquired infections, pressure sores, and GI symptoms due to immobilization. However, blood transfusions are also associated with prolonged postoperative length of stay and increased morbidity, reflecting underlying medical comorbidities and surgical complexity in the patients who require blood transfusions [9].

The threshold value of Hb to begin transfusion is debated. Some studies report that transfusions are associated with an increased risk of postsurgical complications and long-term mortality. Knowledge of how much Hb is lost would help in the appropriate use of blood transfusions in patients who need them. It is acknowledged that most patients undergoing elective surgery with an Hb level of 10 g/dL and above may have allowable blood loss. There is no reason to expose patients to blood transfusions for those who do not need them, which will reduce the risk of reactions as well as infections related to blood transfusions. The aim of this study was to assess the Hb drop and its associated factors among elective orthopedic surgeries at Kilimanjaro Christian Medical Centre (KCMC).

## 2. Methods

### 2.1. Study Design and Setting

This was an analytical cross-sectional study done between October 2022 and March 2023, conducted in the Department of Orthopedics and Trauma at KCMC. The hospital is one of the regional referral hospitals located in the northern zone, at the base of Mt. Kilimanjaro, serving over 15 million people. All adult patients undergoing elective orthopedic surgeries at KCMC were included. However, patients undergoing emergency orthopedic surgeries, revision surgeries, required intraoperative transfusions, multiple surgeries, pre-existing hematological conditions, patients on antiplatelets and/or anticoagulants, given tranexamic acid intraoperatively, and diagnostic surgeries were excluded. The term “elective orthopedic surgeries” implies surgeries that are not emergencies at our center and are scheduled in advance. Emergency orthopedic surgeries consist of open fractures, septic arthritis, and compartment syndrome.

### 2.2. Ethical Clearance

Permission to conduct this study was requested from the medical director of KCMC and the Research Ethical Committee of Tumaini University Makumira through the Director of Postgraduate Studies, and ethical clearance no. PG 161/2022 was obtained. The request to perform the study was authorized by the head of the Department of Orthopedics and Trauma at KCMC. All the information was kept strictly confidential, and identification numbers were assigned unlinked to the patient identifiers.

### 2.3. Sample Size Estimation

A minimum sample size for this study was estimated based on a study done at KCMC that looked at surgery statistics by department. Therefore, a hypothetical proportion of 14.5% was used to estimate the sample size by using the Cochran equation, *N*=(*Z*^2^pq)/(*E*^2^). The minimum sample size required at KCMC was 195 patients. A convenience sampling technique was used to identify patients undergoing elective orthopedic surgeries.

### 2.4. Data Collection

Patient details were obtained through an electronic health management system and filled into a statistical package for social science (SPSS) data sheet. The information collected was age, sex, chronic illness, timing of surgery, diathermy use, tourniquet use, duration of surgery, type of surgery, type of anesthesia, and Hb drop. Patients were initially identified based on the type of orthopedic surgery they were going to undergo. Once identified for elective orthopedic surgery, the patient and/or relatives were approached, counseled, and obtained consent before the start of the data collection. A face-to-face interview was conducted with the patient and/or their relatives to gather preoperative details. Biological sample collection was collected with a 5 cc syringe and put into a purple top EDTA vacutainer for Hb sampling, which was run through the Mindray BC 3200 to obtain the results. Their intraoperative details were recorded in a registry by the theater nurse on duty. A blood sample for postoperative hemoglobin was taken 48 hours postoperatively. The preoperative Hb was recorded from the KCMC lab system and was usually taken within 7 days prior to surgery as per KCMC protocol. Each data entry had an ID number and not the name of the patient, to abide by confidentiality. The data stored were password protected on an electronic device.

### 2.5. Statistical Analysis

The collected data were entered into SPSS version 26. Descriptive data were summarized using narration and tables. The Hb drop was presented as the mean of each type of surgery with a one-way ANOVA analysis at a 95% confidence interval. *T*-tests were used to estimate the association of factors against Hb drop and run through Welch and Brown-Forsythe to account for the difference in SD. A probability (*p* value) less than 0.05 was considered statistically significant.

## 3. Results

**Figure 1:**
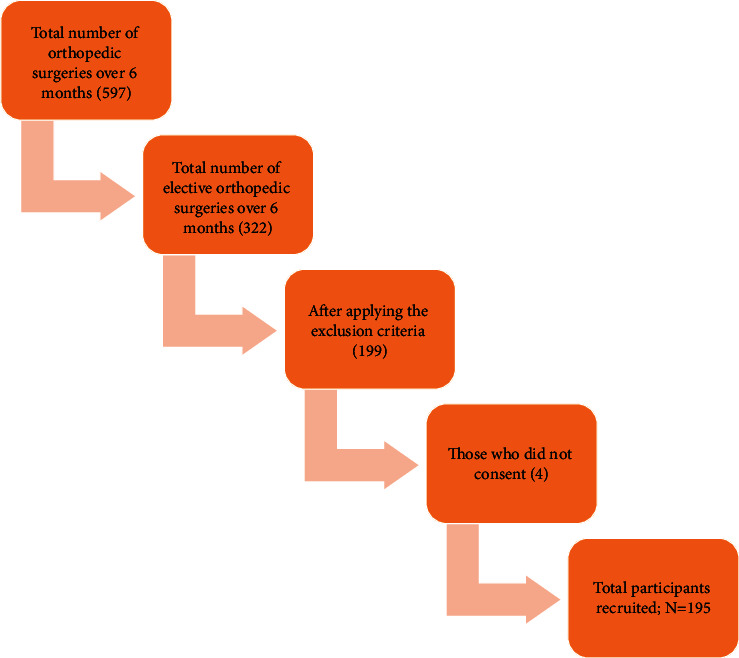
Flowchart demonstrating how the sample was obtained among orthopedic surgeries.

**Figure 2:**
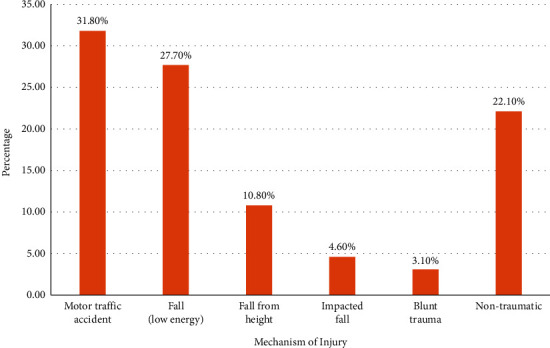
The mechanism of injury among patients undergoing elective orthopedic surgeries (*n* = 195).

A total of 195 participants were enrolled in the study (Figure 1). Of these, 62.1% were males, 73.8% were aged between 18 and 65 years, and 52.8% were insured, as shown in Table 1. Males (62.1%) accounted for almost two thirds of the total number of participants. This shows correspondence to the normal demographic distribution of orthopedic cases, with the ages of the participants showing that younger adults aged 18–65 years (73.8%) were almost three times the number of older adults (26.2%). The majority of the participants were from the region of Kilimanjaro (71.3%), followed by the nearby Arusha region (24.1%), with only one foreigner from Mombasa, Kenya (0.5%). The mechanism of injury was almost equal between motor traffic accidents (MTA) (31.8%) and low energy falls (27.7%), followed by nontraumatic (22.1%) and blunt trauma (3.1%) being the least (Figure 2). There was a close distribution between cash patients (47.2%) and insured patients (52.8%).

**Figure 3:**
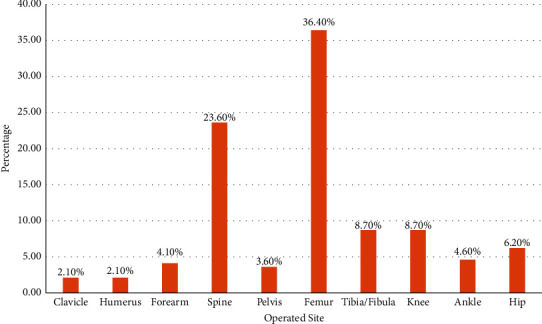
The sites of operation among patients undergoing elective orthopedic surgeries (*n* = 195).

The femur (36.4%) was the most operated single bone in the study, followed by the spine (23.6%), with the clavicle and humerus (2.1%) being the least. It should be noted that the femur is also included in the hip (6.2%) and knee (8.7%) arthroplasty surgeries (Figure 3).

Intramedullary nail (IMN) surgeries (18.5%) were the most popular single type of surgery done in our study, followed closely by decompression and stabilization (16.9%), and least of all, open reduction internal fixation (ORIF) with tension band wiring (TBW), having only two participants (1%).

The mean Hb drop was calculated across the different types of surgeries included in the study, as shown in Table 2. The mean preoperative Hb was measured at 12.85 g/dL (SD: 1.76, 95% CI: 12.60–13.10), with the mean postoperative Hb measuring at 10.10 g/dL (SD: 1.88, 95% CI: 9.84–10.37). Our study showed an overall Hb drop in all surgeries of 2.75 g/dL (SD: 1.47, 95% CI: 2.54–2.96), with the highest losses shown in total hip replacement (THR), followed by IMN. The lowest Hb drop occurred in open reduction and internal fixation with TBW surgeries, followed by upper limb plating and screw surgeries.

One-way ANOVA and *t*-tests (Tables 3 and 4) were used with Hb drop as the dependent variable against different independent variables to analyze for significant association. For diathermy and tourniquet use, only the surgeries that had both values were used. The null hypothesis used was that there was no difference between the categories in terms of Hb drop. The tables summarize the results of the different factors against Hb drop.

The analysis showed no significant differences in the outcome of Hb drop for multiple factors in the study, including age (*p*=0.85), with means almost being similar. The sexes of the participants had similar results with no statistical significance (*p*=0.48). Patients with no comorbidities had a lower mean average than patients with chronic illnesses, which was significant (*p*=0.023). There were differences in mean averages of tourniquet use and type of anesthesia, but they were not statistically significant. There was a significant difference in means of diathermy usage (*p*=0.046). The duration of surgery also showed a significant difference in mean Hb drop (*p*=0.002).

## 4. Discussion

Overall, this study showed a mean Hb drop of 2.75 g/dL. This is higher than in a study done in the United Kingdom (UK), where Hb dropped by 2.48 g/dL [10], and in Nigeria, where the Hb drop was 2.1 g/dL [11]. However, in Uganda, the mean Hb drop was 3.31 g/dL [12], which was significantly higher than our study. In addition, another study in the UK revealed a significant drop in Hb concentration by 2.9 g/dL at day one following surgery and 3.3 g/dL at day two [13]. This shows that regardless of the region, the Hb drop can be attributed to the type of surgery, equipment, or a difference in demographics.

Spine surgeries had a mean Hb drop of 2.73 g/dL, among the lowest levels of deviation. This is close to a study done in China [14], which showed a mean Hb drop of 2.60 g/dL. As these surgeries were all operated by the same specialist at our setting, this could explain the consistent levels of mean Hb drop measured across the surgeries. There was also consistency in regards to the approach, preparation, and implants used across the surgeries, as well as the guaranteed use of diathermy, type of anesthesia, duration of surgery, and blood loss during surgery.

The most common surgeries done were IMN surgeries, which had a mean Hb drop of 3.46 g/dL and were among the highest Hb drops in our study, which correlates to a study done in Uganda among IMN surgeries that had a drop of 3.33 g/dL [12]. The higher SD (1.79) does show a notable difference between the measurements in multiple surgeries. The IMN is one of the first surgeries that residents are trained in during residency, and there are also some specialists who perform the surgery. This could explain the higher range of Hb drop in the particular category of surgeries. The higher Hb drop can be attributed to the fact that all our IMN were ORIFs due to delays in presentation and payment. Studies have shown that closed reduction and internal fixation with IMN are associated with lower Hb loss [15].

Most of the plates in the study were done in the extremities, resulting in a lower mean Hb drop overall than other surgeries. Less soft tissue coverage in the upper limbs, tibia plateau, and ankle areas requires less hemorrhagic stripping than well-covered areas, which is consistent with a higher Hb drop [3]. These surgeries are also consistent with a shorter duration of procedure time compared to the others.

Dynamic hip screw (DHS) had a mean drop of 3.31 g/dL, which correlates with a study done in the UK that showed a mean Hb drop of 3.17 g/dL in DHS surgeries [10]. The procedures are also associated with a higher duration of surgeries, involving opening the fracture site, attaining reduction, inserting derotational wires, insertion of the femoral neck screw, and fixation of the plate, therefore explaining the higher mean Hb drop. This is also reflected in the lower values from the other hip surgeries documented in the study.

The highest mean Hb drop in the study was found to be 4.19 g/dL from THR surgeries. This was higher than shown by a study done in Denmark [16], which had a mean Hb drop of 2.80 g/dL for posthip arthroplasty patients. THR surgeries are associated with a higher Hb drop due to several factors, most notably osteotomy and excision of a highly vascularized part of bone.

Total knee replacement also showed an increase in a study done among knee arthroplasty patients in Taiwan [17], with 3.34 g/dL compared to 2.8 g/dL postoperatively. As reported above, replacement surgeries are expected to have a higher Hb drop as they deal with highly vascularized bone structure and are associated with osteotomies and excisions.

Proximal femoral nail (PFN) surgeries showed a lower mean Hb drop (2.40 g/dL) as compared to hip hemiarthroplasties (2.65 g/dL), which differs from a study done in the UK [18], with cephalomedullary nailing having a higher Hb drop (3.22 g/dL) than hemiarthroplasties (2.09 g/dL). It is expected to have a lower Hb drop in hemiarthroplasty patients as these surgeries mostly do not require intraoperative reduction of fracture fragments, as is essential in PFN. The difference between the two studies might be affected by the surgical approach used, the type of implant, and the surgical tools used in the surgery. Most of the plates in the study were done in the extremities, resulting in a lower mean Hb drop overall than other surgeries. Less soft tissue coverage in the upper limbs, tibia plateau, and ankle areas requires less hemorrhagic stripping than well-covered areas, which is consistent with a higher Hb drop [3]. These surgeries are also consistent with a shorter duration of procedure time compared to the others.

Although the mean Hb drop for males (2.81 g/dL) was slightly higher than in females (2.66 g/dL), which correlated with other studies done in China [19] and Saudi Arabia [20], and the results from our study were not significant (*p*=0.48). There is a wider range of surgeries that male participants underwent compared to their female counterparts in the study, as most females underwent hip and spine surgeries while men had more upper limb and ankle plates and screws. This could explain the similarities in the mean Hb drop between the two groups.

There was also no particular difference in mean Hb drop between younger adults and older adults, which was supported by significance (*p*=0.85). This was different from studies done elsewhere, which showed a significant difference between the two groups in the USA [21] and Iran [22]. As of the time of the study, most of the elderly were involved in hip surgeries, which are associated with a higher Hb drop than extremity surgeries. However, the younger adults were also in surgeries associated with higher Hb drop, mainly IMN and femoral hip screws, which evened out the means between the two groups.

We also found a significant difference (*p*=0.002) between the means of shorter surgeries and longer surgeries, which is similar to a study done in Nigeria [11]. The shorter intraoperative time signifies a shorter time a wound is open after incision, with muscle stripping known to cause significant levels of blood loss. The sooner a surgeon can close up, the more effective they are at reducing blood loss and Hb drop as a whole.

The use of diathermy to cut and coagulate smaller bleeding vessels has largely been associated with lower blood loss in recent times, with our study confirming the significant difference in similar cases between those that used diathermy and those that did not (*p*=0.046). A study done among hemiarthroplasties in Ireland [23] and another done in Uganda [12], specifically in femoral fractures, also had the same findings.

Our study found no significant difference in the mean Hb drop in patients who used tourniquets during surgery (*p*=0.39) even though the means showed a notable difference. This correlated to a study done in Saudi Arabia that found no difference in the use of tourniquets in TKR surgeries [20]. The use of tourniquets has largely been associated with lower levels of bleeding in patients intraoperatively [24], contrary to the results of our study. Some of the surgeries were associated with longer intraoperative duration, which might have offset the mean Hb drop results. However, not all surgeries have the capability of using tourniquets, such as for spine, hip, and femur surgeries, which were not included, and a smaller sample was used in the analysis.

There was no significant difference (*p*=0.08) in mean Hb drop between regional anesthesia and GA even though the mean showed GA having a lower mean compared to regional anesthesia. This differed from a study done in Taiwan [17] in a retrospective study that showed patients who underwent spinal anesthesia were less likely to lose blood than patients undergoing GA. Regional anesthesia is more often associated with less blood loss, with the sympathetic nerve blockage causing less blood oozing. At our center, spinal and upper limb surgeries are more often than not the only surgeries where patients undergo GA, with almost all lower limb surgeries undergoing regional anesthesia. The higher mean blood loss in hip and femur shaft surgeries could explain this difference.

Our study found some changes in the mean Hb drop between patients with no comorbidity and those with chronic illnesses (*p*=0.023). This result was similar to a study done in Poland [25], which tried to find a correlation between patients with comorbidities and the need for blood transfusion. Hypertension is associated with increased blood loss interoperatively due to increased destruction of hemostatic plugs, while diabetes mellitus often causes sluggish blood flow and an increase in circulating proteins, which affects platelet aggregation.

The strength of this study was its ability to provide a baseline on Hb drop for multiple elective procedures performed at our center. There was a lot of diversity among the participants and surgeries performed. However, the study would have benefited from being more focused on a single aspect of correlation as opposed to multiple factors, therefore keeping some aspects constant. In addition, this was a single-center hospital-based study, which may not be representative of the burden and characteristics of stroke cases in the whole population.

## 5. Conclusion

The average decrease in Hb levels across all surgeries was found to be 2.75 g/dL. Accounting for differences in types of surgery, there should not be delays in patients who have a preoperative Hb level that can sustain the mean Hb drop recorded in the study. No significant differences in Hb drop were observed based on gender, age, or the presence of comorbidities, and as such, these factors should not be considered in the preoperative Hb cutoff for eligibility for surgery. However, the application of diathermy and shorter duration of surgeries demonstrated a significant decrease in Hb drop and should be incorporated to minimize Hb drop and blood loss as a whole in orthopedic surgeries.

## Figures and Tables

**Table 1:** Demographic characteristics of participants who underwent elective orthopedic surgeries (*n* = 195).

Descriptive variable	*n* (%)
Sex	
Male	121 (62.1)
Female	74 (37.9)
Age (years)	
18–65	144 (73.8)
≥66	51 (26.2)
Region	
Kilimanjaro	139 (71.3)
Arusha	47 (24.1)
Others	9 (4.6)
Form of payment	
Cash	92 (47.2)
Insured	103 (52.8)

**Table 2:** Mean Hb drop across different elective orthopedic surgeries (*N* = 195).

Type of surgery	*n*	Mean (g/dL)	SD	95% CI for mean		*p* value
				Lower	Upper	
Decompression + stabilization	46	2.73	0.91	2.46	3.00	<0.001
THR	12	4.19	1.37	3.32	5.06	<0.001
TKR	17	3.35	1.34	2.66	4.04	<0.001
Hemiarthroplasty	16	2.66	1.59	1.81	3.50	<0.001
Acetabular recon (plate and screws)	7	2.06	0.47	1.62	2.49	0.004
Plating and screws (upper limb)	15	1.43	0.77	1.01	1.86	0.009
Ankle recon (plate and screws)	10	1.70	1.18	0.86	2.54	0.021
IMN	36	3.46	1.79	2.86	4.07	<0.001
DHS	6	3.32	1.56	1.68	4.95	0.004
PFN	18	2.40	1.35	1.73	3.07	<0.001
Tibia plateau ORIF	10	1.86	1.14	1.04	2.68	0.008
ORIF with TBW	2	0.85	0.35	−2.33	4.03	0.79
Total	195	2.75	1.48	2.54	2.96	<0.001

THR: total hip replacement; TKR: total knee replacement; IMN: intramedullary nail; DHS: dynamic hip screw; PFN: proximal femoral nail; ORIF: open reduction internal fixation; TBW: tension band wiring.

**Table 3:** One-way ANOVA and *t*-tests with Hb drop as independent variables against preoperative factors among elective orthopedic surgeries (*N* = 195).

	*n*	Mean (g/dL)	*F*	*p* value
Age (years)				
18–65	144	2.748	(1, 193) 0.038	0.85
≥66	51	2.794		
Sex				
Male	121	2.817	(1, 193) 0.492	0.48
Female	74	2.666		
Duration of symptoms (weeks)				
≤2	90	2.563	(1, 193) 3.071	0.08
>2	105	2.929		
Chronic illness				
Yes	38	3.313	(1, 193) 6.997	0.023
None	157	2.676		

**Table 4:** One-way ANOVA and *t*-tests with Hb drop as independent variables against intraoperative factors among elective orthopedic surgeries (*N* = 195).

	*n*	Mean (g/dL)	*F*	*p* value
Duration of surgery (hours)				
≤2	98	2.435	(1, 193) 10.267	0.002
>2	97	3.089		
Diathermy use				
Yes	41	2.190	(1, 96) 3.545	0.046
No	57	2.812		
Tourniquet use				
Yes	29	1.848	(1, 59) 0.764	0.39
No	32	2.134		
Type of anesthesia				
General	65	2.534	(1,193) 2.360	0.08
Regional	130	2.873		

## Data Availability

The datasets generated during and/or analyzed during the current study are available from the corresponding author on reasonable request.
